# Ras1-Independent High Iron-Mediated Hyphal Formation in *Candida albicans*

**DOI:** 10.3390/jof12070459

**Published:** 2026-06-23

**Authors:** Deepak Parashar, Rishabh Sharma, Sumant Puri

**Affiliations:** Oral Microbiome Research Laboratory, Temple University, Philadelphia, PA 19140, USA

**Keywords:** *C. albicans*, iron, hyphae, Ras1, cAMP-Protein Kinase A signaling

## Abstract

*C. albicans* small GTPase Ras1 belonging to the cAMP-Protein Kinase A (PKA) signaling pathway is a well-established master regulator of hyphal development, taking its environmental cues from N-acetylglucosamine (GlcNAc) as a carbon source. Iron is also known to induce filamentation in *C. albicans*. However, the influence of iron availability on Ras1-cAMP-PKA signaling in response to GlcNAc-induced filamentation has never been studied. In this study, we investigated the role of Ras1 in hyphal induction under varying iron conditions, using both in vitro systems and an in vivo model of mucosal colonization in *Caenorhabditis elegans*. Surprisingly, upon GlcNAc exposure, *Δ/Δras1* cells formed true hyphae exclusively under high-iron conditions, whereas its parent strain (CAI4-Ura+) showed hyphal formation irrespective of environmental iron levels. Further analysis revealed that this GlcNAc-mediated hyphal formation under high iron in *Δ/Δras1* cells was independent of cAMP levels but required the downstream effectors Efg1 and Tpk2. A similar iron-dependent pattern of hyphal formation in *Δ/Δras1* cells was also observed in vivo in *C. elegans*. Transcriptomic analysis indicated that high iron induced robust expression of hypha-associated genes in *Δ/Δras1*, accompanied by downregulation of *BCY1*, a negative regulator of PKA. Overexpression of *BCY1* in *Δ/Δras1* cells completely blocked the iron-dependent hyphal formation, highlighting a previously unrecognized Ras1-independent, iron-responsive mechanism controlling PKA-mediated filamentation. Collectively, our findings reveal that increased environmental iron availability can bypass Ras1 to regulate hyphal development by limiting Bcy1 levels to allow PKA activation. This provides insights into how *C. albicans* can exploit iron replete host niches for enhanced pathogenicity, eliminating the need for key modulators such as Ras1.

## 1. Introduction

*Candida albicans* is a commensal fungus that colonizes the oral cavity and the gastrointestinal tract, as well as various other epithelial surfaces including the skin and vaginal mucosa of healthy individuals [1]. However, disruptions in host immunity or microbiota can lead to its overgrowth, resulting in infections ranging from superficial candidiasis to life-threatening systemic infections [2]. *C. albicans* exhibits two primary morphological forms: the unicellular yeast form and the filamentous hyphal form. The yeast form is typically associated with commensal colonization, while the hyphal form is linked to tissue invasion and pathogenicity [3]. This morphological switch is triggered by environmental cues such as pH, temperature, carbon sources such as N-acetylglucosamine (GlcNAc), or metals like iron [4,5].

GlcNAc is a monosaccharide sugar that plays a crucial role in the growth, morphogenesis, and pathogenicity of *C. albicans* [6]. The human oral cavity, in particular, contains glycoproteins such as mucins, salivary glycoproteins, and epithelial cell surface glycoproteins that release GlcNAc upon degradation by microbial or host enzymes [7]. In the absence of glucose, *C. albicans* can utilize GlcNAc as both a carbon and nitrogen source for metabolism [8]. One of the most critical roles of GlcNAc in *C. albicans* infections is its ability to induce hyphal formation, a key virulence factor in oral candidiasis [6]. GlcNAc activates signaling pathways involved in filamentation, specifically the cAMP-PKA pathway that activates transcription factor (TF) Efg1, leading to hyphal specific gene expression, as well as the Mitogen-activated Protein Kinase (MAPK) pathway TF Cph1 that is involved in cell wall remodeling and invasion [9,10].

**Figure 1:**
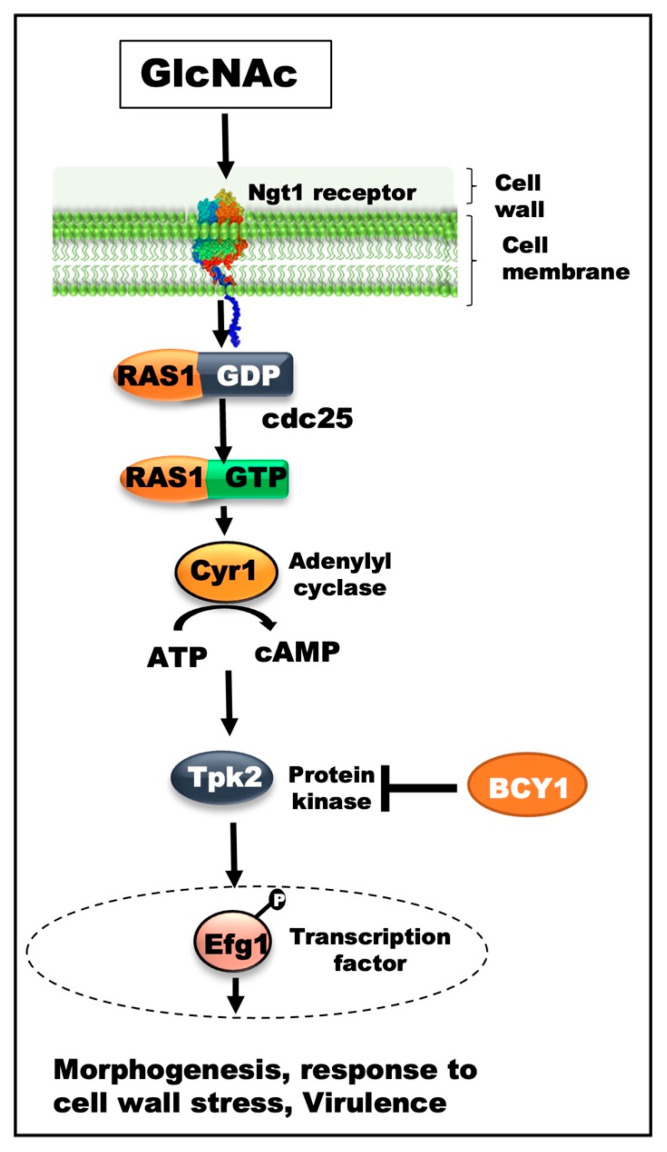
Model of GlcNAc-induced Ras1-dependent signaling during hyphal morphogenesis in *C. albicans*. Schematic representation of the canonical Ras1-cAMP-PKA signaling pathway regulating hyphal formation in response to GlcNAc stimulation. Under hypha-inducing conditions, Ras1 activates the adenylate cyclase Cyr1, leading to increased intracellular cAMP production and subsequent activation of the PKA pathway, which promotes hyphal growth and morphogenesis. Bcy1 functions as the negative regulatory subunit of PKA and suppresses downstream PKA signaling under non-inducing conditions. Reduction of BCY1 expression relieves inhibition of PKA activity and facilitates hyphal development downstream of the Ras1–cAMP signaling cascade.

The cAMP–PKA signaling pathway is a key regulator of morphogenesis in *C. albicans* and is highly conserved across eukaryotes. It is activated by the small GTPase Ras1, which stimulates the adenylyl cyclase Cyr1 to generate cAMP [11]. Elevated cAMP binds to the regulatory subunit Bcy1, relieving inhibition of the PKA holoenzyme and releasing the catalytic subunits Tpk1 and Tpk2 [12,13]. These kinases phosphorylate downstream targets, including transcription factors such as Efg1, to drive hyphal specific gene expression and morphological transitions essential for virulence [14] (Figure 1). Loss of Ras1 impairs cAMP production and hyphal development [11]. In parallel, Ras1 also activates the Cek1 MAPK pathway, which regulates filamentation, cell wall remodeling, and adhesion [15]. Together, these pathways coordinate morphological responses and contribute to *C. albicans* pathogenicity.

Iron, another filamentation-inducing signal in *C. albicans* [5], is essential for all organisms, including fungi, as it serves as a cofactor in many cellular processes such as respiration, DNA synthesis, and oxidative stress response [16]. To restrict microbial access to iron, the host employs a defense strategy known as nutritional immunity, wherein free iron is tightly sequestered by proteins such as transferrin, lactoferrin, and ferritin [17]. As a result, pathogenic microbes developed specialized strategies to acquire iron within the host. *C. albicans* is equipped with multiple iron acquisition systems and iron-protective pathways, enabling it to thrive in niches with varying iron levels, including the iron-rich oral cavity [18,19,20]. Additionally, iron availability can modulate key virulence traits in *C. albicans* [21,22].

We previously demonstrated that in the presence of glucose as carbon source, iron can activate the *C. albicans* Cek1 pathway to affect cell wall architecture [23,24]. However, how iron availability influences the Ras1-cAMP-PKA pathway in the presence of GlcNAc as a carbon source remains unclear. To address this, we investigated here how *C. albicans* cells behave in response to changes in environmental iron levels when GlcNAc is present as the sole carbon source, using *C. albicans* cells lacking Ras1 (*Δ/Δras1*), along with its parent strain.

## 2. Materials and Methods

### 2.1. Fungal Strains, Media, and Culture Conditions

*C. albicans* strains used in this study are listed in Table 1. On day 1, all strains were grown at 30 °C in a basal YNB glucose-based medium lacking copper, ammonium salt, and iron. The medium was supplemented with 2.5 µM CuSO_4_, 5 g/L NH_4_SO_4_, 2% glucose, and 0.79 g/L amino acid supplement (Complete Supplement Mix; 4500-012, MP Biomedicals, Irvine, CA, USA), along with 50 µM of the iron chelator bathophenanthroline disulfonic acid (BPS; 146617, Sigma-Aldrich, St. Louis, MO, USA). For high- and low-iron conditions, ferric chloride was added to this medium to achieve defined iron concentrations of 2 µM (low iron) and 100 µM (high iron), respectively.

For experiments involving hyphal-inducing conditions, exponential-phase cells obtained from the day 1 overnight cultures above were harvested and transferred to hyphal induction medium (YNB-GlcNAc), in which glucose was replaced with 1.2% GlcNAc, under the respective high- and low-iron conditions. The cultures were inoculated at an initial OD_600_ of 0.3 and incubated at 37 °C for 6 h with shaking at 200 rpm. Following incubation, cells were collected and examined microscopically.

### 2.2. Assessment of Hyphal Development

Hyphal formation was assessed by measuring hyphal length using an EVOS M5000 Imaging System (Invitrogen, Waltham, MA, USA), and image analysis was performed using the integrated EVOS M5000 software (Invitrogen). For better resolution, hyphae were also imaged using an inverted Leica TS5 confocal microscope (Leica Microsystems, Wetzlar, Germany). *C. albicans* cells were also stained with Calcofluor White (5 μg mL^−1^) and examined under a fluorescence microscope to visualize hyphal structures based on bright chitin-specific fluorescence [29].

### 2.3. Scanning Electron Microscopy (SEM) of C. albicans Cells

*C. albicans* cells grown in hyphal-inducing conditions were also fixed with 4% glutaraldehyde in 0.1 M phosphate buffer (pH 7.2) for 2 hrs at a low temperature (4 °C) on silicon wafers. Samples were then washed with 0.1 M phosphate buffer (pH 7.2). Post-fixation was carried out for 2 h at 4 °C. Cells were then rinsed with 0.1 M phosphate buffer (pH 7.2). After washing, cells were dehydrated in a series of ethanol gradients: 30%, 50%, 70%, 90%, and 100% (each for 10 min). Finally, samples were imaged with a scanning electron microscope (FEI Quanta 450FEG, FEI Company, Hillsboro, OR, USA) at ×8000 magnification [30].

### 2.4. C. albicans Growth Profile

To evaluate *Δ/Δras1* cell growth under varying iron conditions, *C. albicans* cells were grown in a YNB-glucose medium under low- and high-iron conditions overnight at 30 °C, with shaking at 200 rpm. The following day, overnight cultures were diluted in fresh high- and low-iron YNB-glucose medium at an initial optical density (OD_600_) of 0.1. A total volume of 200 µL of the diluted cell suspension was dispensed into each well of a 96-well polystyrene microtiter plate (Cat. No. 655180, Greiner Bio-One, Kremsmünster, Austria). The plate was incubated at 30 °C, and growth kinetics were monitored by measuring absorbance at 600 nm at 30 min intervals with 5 s plate shaking over a 24 h period using a BioTek Synergy Multi-Mode microplate reader [20].

### 2.5. C. elegans Infection Assay

Synchronized populations of wild-type N2 *C. elegans* were obtained using a standard bleaching method [31]. Briefly, adult worms were collected from nematode growth medium (NGM) agar plates and treated with a freshly prepared alkaline hypochlorite (bleach–NaOH) solution to dissolve adult bodies while preserving eggs. The recovered eggs were washed three times with a sterile M9 buffer and incubated in M9 buffer without a food source at 16 °C to allow synchronous hatching. Under these conditions, L1 larvae were developmentally arrested, yielding a synchronized population. Synchronized L1 larvae were then transferred to NGM plates seeded with the *E. coli* OP50 for subsequent experiments to obtain adult worms. Once worms reached adult stage, they were harvested and washed with M9 buffer to remove any residual bacterial cells. Approximately ~40 worms/well were transferred in six-well plates containing low- and high-iron YNB-GlcNAc media, and infected with *C. albicans* cells (either *Δ/Δras1* or its parent strain) at an initial optical density (OD_600_) of 0.1. Worms were then monitored each day using an inverted microscope and percent survival of worms was calculated on day 6 of incubation. For visualization of *C. albicans* filamentation within the host, worms were mounted on glass slides and examined using an EVOS M5000 Imaging System (Invitrogen, Waltham, MA, USA).

### 2.6. cAMP Measurement

*C. albicans* cells grown under hyphal inducing conditions were used to measure intracellular cAMP levels using the cAMP-Screen Direct Immunoassay System (Applied Biosystems, Foster City, CA, USA), following the manufacturer’s instructions. Briefly, cells were lysed to release intracellular contents by using the acid-washed glass beads for 5 cycles of 1 min vortexing in FastPrep^®^-24 instrument (MP Biomedicals, Irvine, CA, USA), with samples cooled on ice for 1 min between cycles. Lysed cells were centrifuged at 10,000× *g* for 5 min at 4 °C. After centrifugation, supernatant was collected and protein estimation was performed using Pierce BCA Protein Assay Kit (A65453, Thermo Fisher Scientific, Rockford, IL, USA), 20 µg of respective samples were added to the assay plate along with cAMP detection reagents, and competitive immunoassay was performed as per kit instructions.

### 2.7. Transcriptomic Analysis

Total RNA was isolated from *C. albicans* cells were grown under hyphal-inducing conditions, using the RNeasy Mini Kit (Qiagen, Hilden, Germany). Briefly, cells harvested from 10 mL cultures were washed with PBS and resuspended in 350 μL of RLT lysis buffer. Mechanical disruption was performed using 0.45 mm glass beads in a FastPrep-24 homogenizer (MP Biomedicals) at 6 m/s for six to seven cycles. Following centrifugation to remove cellular debris, supernatants were processed for RNA purification according to the manufacturer’s instructions. Residual genomic DNA was eliminated using the TURBO DNA-free™ kit (Invitrogen), and RNA purity was evaluated by measuring the A260/A280 ratio using a NanoDrop spectrophotometer (Thermo Fisher Scientific).

Strand-specific mRNA libraries were prepared and sequenced on the Illumina NovaSeq X Plus platform using paired-end 150 bp reads. Each sample yielded more than 20 million read pairs with Q30 scores exceeding 85%. Raw sequencing data (approximately 6 Gb/sample) were aligned to the *C. albicans* reference genome (GCF_000182965_3_asm18296v3; taxon 5476) using HISAT2 (v2.0.5). Read quantification and normalization were carried out in Strand NGS using the DESeq2 pipeline, with differential expression analysis performed using Benjamini–Hochberg correction to control the false discovery rate (FDR q < 0.05). Library preparation, sequencing, and primary bioinformatics processing—including quality control, genome alignment, normalization, and differential expression analysis—were conducted by Novogene (Sacramento, CA, USA) following standard protocols. Genes with an adjusted *p*-value ≤ 0.05 and an absolute log_2_ fold change ≥ 1 were considered significantly differentially expressed. They were then ranked by fold change and used as input for iDEP 2.01 [32] to generate heat map and pathway enrichment analysis.

### 2.8. Real Time Quantitative PCR

RNA samples generated as above were separately also treated with DNase to remove any DNA contamination by using the TURBO DNA-free™ Kit (Thermo Scientific). One microgram of total RNA was reverse-transcribed into cDNA using the iScript™ cDNA Synthesis Kit (Bio-Rad, Hercules, CA, USA, #1708891). Quantitative PCR was performed using SYBR Green Supermix (Bio-Rad, #1725124) on a QuantStudio 3 Real-Time PCR System with gene-specific primers. Relative transcript levels were normalized to GAPDH RNA expression and calculated from standard curves. Results represent the mean ± SEM from three independent experiments.

### 2.9. Generation of Bcy1-Overexpressing Δ/Δras1 Strain

*BCY1* gene was amplified using gene-specific forward and reverse primers (with the reverse primer designed to carry overhang 6x His-tag (CATCACCATCACCATCACTAA)). Plasmid pNIM1 and the PCR product (denaturation at 95 °C, annealing at 60 °C, extension at 72 °C) of the above primer set were digested with *Bgl*II and *Sal*I, and the resulting fragments were ligated, following the manufacturer’s protocol (Thermo Scientific). The ligation product was transformed into *E. coli* DH5α and plated on LB-amp plates (100 µg/mL). Ampicillin resistant colonies were screened using *BCY1* gene-specific primers, and one positive clone was grown for large-scale plasmid preparation. A recombinant *Δ/Δras1* strain was constructed by introducing the recombinant pNIM1-*BCY1* cassette by electroporation, as previously described [33]. Briefly, 5 µg of DNA was transformed into 50 µL of competent *Δ/Δras1* cells and electroporated in a 0.2 cm cuvette at 1.8 kV using the Gene Pulser Xcell™ system (Bio-Rad, USA). After electroporation, cells were resuspended in 1 mL of ice-cold 1 M sorbitol and transferred to a collection tube. Cells were collected by centrifugation at 4000 rpm, resuspended in 1 mL YPD medium, and incubated at 30 °C for 4 h. Cells were then plated on YPD plates supplemented with tetracycline and incubated at 30 °C for 2 days. Transformants were selected on YPD agar plates supplemented with 100 μg/mL tetracycline and 100 μg/mL nourseothricin and incubated at 30 °C for 2 days. Positive transformants were screened by colony PCR using *BCY1*-specific primers, and *BCY1* overexpression was confirmed by RT-PCR (Table 2). For induction of BCY1 expression, doxycycline was added to the culture medium at a final concentration of 20 μg/mL.

### 2.10. Statistical Analysis

Statistical analysis was performed using one-way ANOVA or unpaired Student’s t-test as indicated, between the low- and high-iron study groups, using GraphPad Prism software version 10.4.2.

## 3. Results

### 3.1. C. albicans Δ/Δras1 Cells Formed GlcNAc-Induced Hyphae Under High Iron

**Figure 2:**
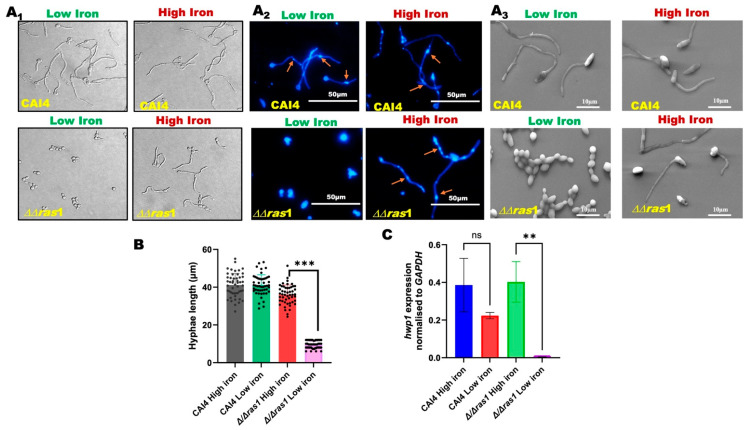
GlcNAc-induced hyphal formation in CAI4 and *Δ/Δras1* strains under low and high iron. (**A**) *C. albicans* CAI4 and *Δ/Δras1* strains were grown in YNB-GlcNAc under low- and high-iron conditions to study morphological changes. (**A_1_**) Micrograph of unstained live *C. albicans* cells, (**A_2_**) Calcofluor White-stained *C. albicans* cells, and (**A_3_**) high-resolution analysis of *C. albicans* cells by scanning electron microscopy (SEM). (**B**) Hyphal length quantification was performed using images from (**A_1_**), with at least 50 cells analyzed per condition (n ≥ 50). Data shown are representative of three independent biological replicates. (**C**) Relative expression levels of the hyphal specific gene *HWP1* in CAI4 and *Δ/Δras1* strains, determined by qPCR under the corresponding iron conditions. Data are presented as mean ± SEM from three independent experiments. Statistical significance analysis was assessed by one-way ANOVA; ** *p* < 0.01, *** *p* < 0.001, ns: statically not significant.

*C. albicans* parent strain exhibited GlcNAc-induced hyphal formation under both low (2 µM) and high (100 µM) iron. In contrast, *Δ/Δras1* cells formed hyphae only under high iron (Figure 2A_1_). To confirm whether this is not related to any potential growth defects in *Δ/Δras1* strain under low iron, we assessed the growth of *Δ/Δras1* and its parent strain in our YNB-based minimal medium under low and high iron at 30 °C. Both strains showed similar growth profiles under both iron conditions (Figure S1), ruling out growth defect as the reason for lack of hyphal induction for *Δ/Δras1* cells under low iron.

To confirm if hyphae observed in *Δ/Δras1* cells under high iron represent true hyphae, the cells were stained with Calcofluor White to detect cell wall chitin. Similarly to the parent strain under both iron conditions, hyphae formed by the *Δ/Δras1* cells under high iron displayed the first septum distal to the bud neck (Figure 2A_2_), a characteristic feature of true hyphae [34]. Further examination of hyphae using SEM showed that high iron *Δ/Δras1* cells formed a continuous, flattened cell wall at septa without constriction (unlike pseudohyphae which shows neck-like constrictions) [3,30], similar to the hyphae made by its parent strain, under low and high iron (Figure 2A_3_). In addition, hyphal length quantification from Figure 2A_1_ showed that the *Δ/Δras1* cells grown under high iron showed hyphal lengths similar to that of hyphae made by the parent strain cells under both low and high iron (Figure 2B).

Consistent with the above observations, expression analysis of hyphal-specific *HWP1* demonstrated a significant increase in *HWP1* transcript levels in the *Δ/Δras1* cells under high iron compared with low iron, whereas the parent strain showed no significant difference in *HWP1* expression between the two iron conditions (Figure 2C). Together, these results demonstrate that *Δ/Δras1* cells are able to make true hyphae when environmental iron is elevated.

### 3.2. Δ/ΔRas1 Cells Form Hyphae in a High-Iron Host

**Figure 3:**
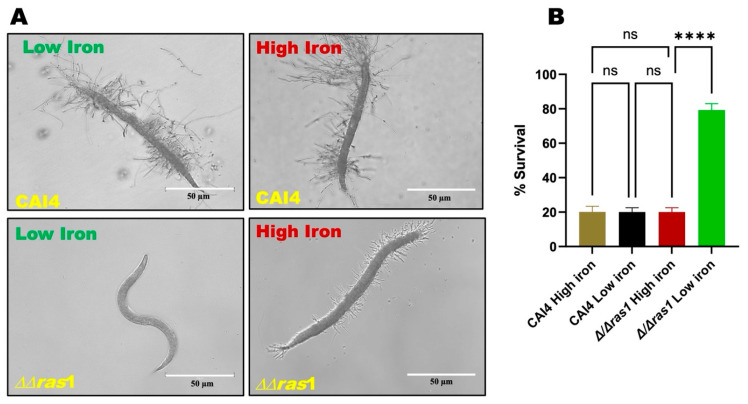
Hyphal development occurs in *Δ/Δras1* mutant under elevated host iron. (**A**) Micrograph showed iron-dependent hyphal formation of *Δ/Δras1* and its parent strain in liquid killing assay of *C. elegans*. Worms infected with the parental CAI4 strain showed robust hyphal formation regardless of iron levels, whereas the *Δ/Δras1* mutant formed hyphae only under high-iron conditions. Scale bars: 50 µm. (**B**) Percentage survival of *C. elegans* after infection with *Δ/Δras1* and its parent strain under low and high iron was calculated on day 6 of incubation. Data represent mean ± SEM. Statistical significance analysis was assessed by one-way ANOVA; (**** *p* < 0.0001, ns: statically not significant).

Environmental iron availability can alter iron accumulation and iron-responsive gene regulation in *C. elegans* [35], indicating that host iron homeostasis is influenced by external iron conditions. To evaluate the iron-dependent hyphal formation of *Δ/Δras1* strain in vivo, we next tested the ability *Δ/Δras1* cells to infect *C. elegans* under both low and high iron. Worms infected with the parent strain exhibited robust hyphal formation in worms (Figure 3A), irrespective of the iron conditions. Similarly to in vitro results (Figure 2), *Δ/Δras1* strain demonstrated hyphal formation only in high-iron worms (Figure 3A); however, *Δ/Δras1* hyphae were shorter in length compared to CAI4 hyphae (Figure 3A). Further, highest worm survival rates were observed for low-iron worms infected with *Δ/Δras1* strain (Figure 3B), while high mortality rates were observed in all other conditions. Thus, Ras1-independent, iron-dependent hyphal formation offers an alternative filamentation-induced virulence pathway in a high iron host.

### 3.3. Iron-Dependent Hyphal Formation in Δ/Δras1 Is Independent of cAMP Signaling While Requiring Efg1 and Tpk2

**Figure 4:**
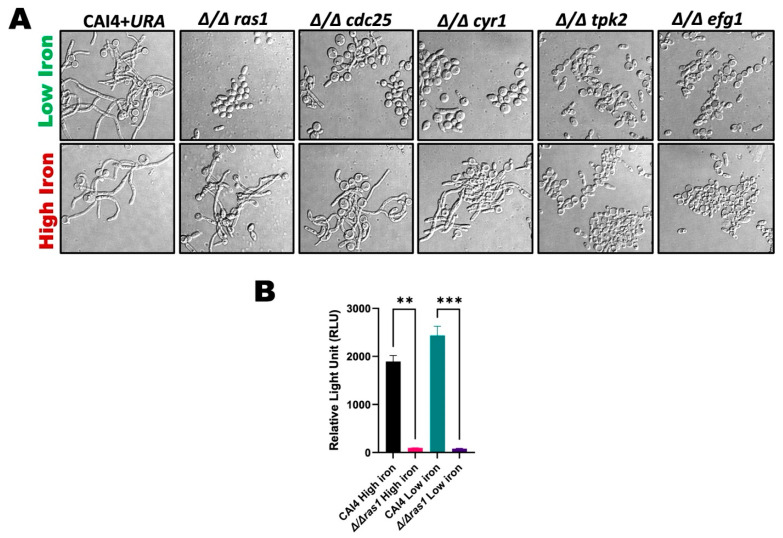
Iron-induced hyphal formation in *Δ/Δras1* occurs independently of cAMP signaling but depends on Efg1 and Tpk2. (**A**) Various mutants of Ras1 pathway strains tested for hyphal formation under low- and high-iron conditions. Among all mutants tested, *Δcdc25* and *ΔΔcyr1* were still able to form hyphae under high-iron conditions. However, *ΔΔtpk2* and *ΔΔefg1* showed a complete block in hyphal formation. (**B**) Intracellular cAMP level was measured under low and high iron in CAI4 and *Δ/Δras1* cells. Data representative of at least two independent replicate experiments and ± standard errors of the means (SEM). Statistical significance analysis was assessed by one-way ANOVA; (** *p* < 0.01, *** *p* < 0.001, ns: statically not significant).

To determine if any of the critical Ras1 pathway proteins play any role in Ras1-independent, iron-dependent hyphal formation, different Ras1 pathway mutant strains (*Δ/Δcdc25*, *Δ/Δtpk2*, *Δ/Δcyr1*, and *Δ/Δefg1*) were tested for GlcNAc-induced hyphal formation under high and low iron. Interestingly, cells lacking Ras1 regulator Cdc25 or the enzyme (Cyr1) required to convert ATP to cAMP retained the ability to form iron-dependent GlcNAc-induced hyphae, similar to *Δ/Δras1* cells (Figure 4A). However, cells lacking downstream protein kinase Tpk2- or Ras1-specific transcription factor Efg1 were not able to produce hyphae in response to GlcNAc under either iron condition (Figure 4A). This suggests that high iron can only bypass the Ras1 pathway for steps involving Ras1 activation by Cdc25 and cAMP formation by Cyr1.

To confirm if high iron alternatively enhances cAMP levels in *Δ/Δras1* cells, we determined cellular cAMP levels in *Δ/Δras1* and its parent strain. Surprisingly, cAMP levels were significantly lower in *Δ/Δras1* cells, compared to CAI4 (Figure 4B), under both iron conditions. Overall, this suggests the involvement of an iron-dependent, cAMP-independent pathway that can compensate for the absence of Ras1 activation and cAMP formation to promote GlcNAc-induced hyphal formation under high iron.

### 3.4. Unique Iron-Responsive Reduction in BCY1 Gene Expression Under High Iron in Δ/Δras1 Cells

**Figure 5:**
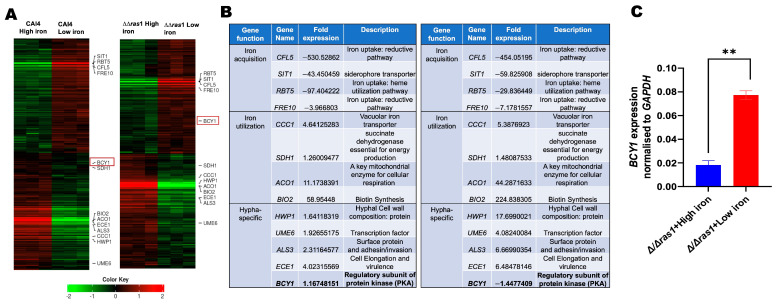
*Δ/Δras1* cells exhibit iron-responsive repression of BCY1. (**A**) Transcriptional profiling of *Δ/Δras1* and the parental strain was performed under low- and high-iron conditions, and heat map shows all differentially expressed genes identified across three biological replicates in *Δ/Δras1* relative to CAI4. Red box showed expression of *BCY1* gene in CAI4 (high iron vs. low iron) and *Δ/Δras1* (high iron vs. low iron). *(***B**) Specific iron and hyphae related differentially expressed genes were analyzed as CAI4 (high iron vs. low iron) and *Δ/Δras1* (high iron vs. low iron). (**C**) Real-time qPCR analysis showed that *BCY1* gene was downregulated in *Δ/Δras1* under low-iron conditions compared to high-iron conditions. Cells were obtained from high-iron and low-iron hyphal induction medium (YNB-GlcNAc), incubated at 37 °C for 6 h with shaking at 200 rpm. GAPDH was used as the house-keeping gene control to normalize gene expression across conditions. The results, which are at least two independent biological repeats in triplicates, are represented as means ± SEM. Statistical significance analysis was assessed by unpaired t-test (** *p* ≤ 0.001, ns: statically not significant).

To investigate the transcriptional basis of iron-induced hyphal formation in the absence of Ras1, we performed transcriptomic analysis of *Δ/Δras1* and its parent strain under hyphal-inducing conditions (YNB + GlcNAc) in low and high iron, using RNA sequencing. Consistent with the expected iron effect on key iron homeostasis genes [23], several iron acquisition genes, including *CFL5, SIT1, RBT5, and FRE10*, were significantly downregulated (Figure 5A,B), while iron utilization and storage-related genes, such as *CCC1, SDH1, ACO1*, and *BIO2*, were significantly upregulated, under high iron compared to low iron in both strains, indicating appropriate transcriptional adaptation to environmental iron. In addition, key hyphal genes, including *HWP1, UME6, ALS3,* and *ECE1*, showed greater upregulation in high vs. low iron *Δ/Δras1* cells compared to its parent strain, reflecting the ability of *Δ/Δras1* strain to undergo filamentation only under high iron.

One striking difference between the two strains was observed for the expression of the regulatory subunit of protein kinase A, Bcy1. This regulatory subunit of the cAMP-PKA pathway suppresses PKA activity and highlights its established role in regulating hyphal morphogenesis in *C. albicans*. Interestingly, *BCY1* was significantly downregulated in the *Δ/Δras1* strain under high iron, compared to low iron, while the parent strain did not show this iron-dependent downregulation of the *BCY1* gene. qPCR analysis of *BCY1* expression corroborated the RNA-seq results, wherein *BCY1* expression was significantly lower in *Δ/Δras1* strain grown under high iron (Figure 5C), compared to low-iron *Δ/Δras1* cells. These results suggest that an iron-mediated decrease in Bcy1 may provide an alternative to cAMP-mediated relief of the inhibitory effect of Bcy1 on Tpk2, thereby allowing GlcNAc-induced hyphal formation despite the absence of active Ras1-cAMP signaling.

### 3.5. BCY1 Overexpression Blocks Iron-Dependent Hyphal Formation in Δ/Δras1 Cells

Bcy1 serves as a key negative regulator of the PKA pathway by restraining Tpk1/Tpk2 activity under non-inducing conditions. Previous studies have demonstrated that modulation or reduction of BCY1 function can bypass defects in upstream Ras1 signaling and promote filamentation through activation of downstream PKA-dependent pathways [13,36].

**Figure 6:**
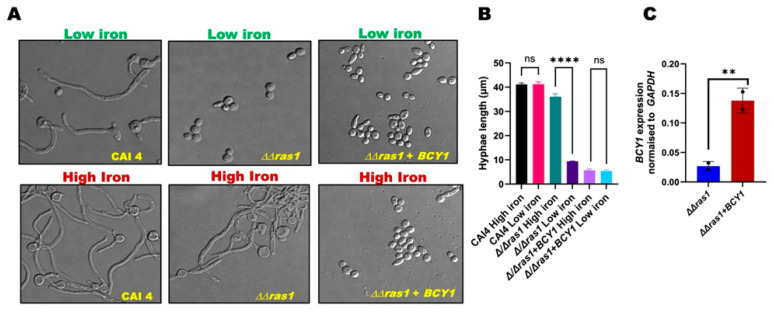
Overexpression of Bcy1 inhibits iron-induced hyphal formation in *Δ/Δras1* cells. (**A**) Morphological comparison of CAI4, *Δ/Δras1*, and *Δ/Δras1+ BCY1* strains were determined in YNB-GlcNAc under high- and low-iron conditions. (**B**) Hyphal length of *C. albicans* and *Δ/Δras1* strains grown under high- and low-iron conditions (no. of cell are n ≥ 50). Data representative of three independent replicate experiments. Statistical significance analysis was assessed by one-way ANOVA; **** *p* < 0.0001, ns: not statically significant. (**C**) *Δ/Δras1* and *Δ/Δras1+ BCY1* strains were grown in YNB-GlcNAc. *Δ/Δras1+ BCY1* strain was induced with 20 µg of doxycycline. Real-time PCR analysis showed overexpression of *BCY1* in *Δ/Δras1+ BCY1* cells compared to *Δ/Δras1* cells. Data representative of at least two independent replicate experiments and ± standard errors of the means (SEM). Statistical significance analysis was assessed by unpaired *t*-test (** *p* ≤ 0.01).

To further investigate whether high iron-mediated downregulation of *BCY1* expression plays a critical role in hyphae formation in the *Δ/Δras1* cells, we overexpressed *BCY1* in the *Δ/Δras1* strain. Successful overexpression of *BCY1*, using pNIM1 expression vector, was confirmed by qPCR, with the *Δ/Δras1* + *BCY1* strain showing significantly higher *BCY1* transcript levels compared to the *Δ/Δras1* strain (Figure 6C). To assess the effect of this overexpression on GlcNAc-induced hyphal formation, the *Δ/Δras1* + *BCY1* strain, along with *Δ/Δras1* and its parent strain, were exposed to GlcNAc for hyphal induction, under both low and high iron, as done previously (Figure 2 and Figure 4). *BCY1* overexpression did indeed completely abolish iron-induced hyphal formation in the *Δ/Δras1* + *BCY1* strain compared to the original *Δ/Δras1* strain (Figure 6A,B), suggesting that iron-mediated downregulation of *BCY1* is responsible for GlcNAc-induced hyphal formation in high-iron *Δ/Δras1* cells.

## 4. Discussion

Morphological plasticity driven by the yeast-to-hyphal switch is important for *C. albicans* pathogenicity [37] and is mainly controlled by the Ras1–cAMP–PKA pathway [38]. This pathway enables *C. albicans* to sense diverse host environmental cues including CO_2_, nutrient, and serum availability [39,40] and drives key virulence attributes such as adhesion, biofilm formation, tissue invasion, and host colonization [41]. Host environments are highly heterogeneous in iron availability, with specific niches such as sites of tissue damage and hemolysis, or in iron-overloaded oral cavity providing elevated iron levels [42,43,44]. How *C. albicans* integrates iron availability with morphogenetic signaling remains incompletely understood.

Here, we demonstrate that *C. albicans* maintained its ability to form GlcNAc-induced hyphae in the absence of Ras1 when supplemented with high iron. Detailed analyses of septal positioning, ultrastructural morphology, hyphal length, and expression of hypha-specific markers such as *HWP1* demonstrated that these hyphae made by *Δ/Δras1* cells in the presence of iron represent true hyphae (Figure 2). Similarly, a recent study has demonstrated that hyphal development in *C. albicans* can be regulated independently of Ras1 signaling in the presence of other specific environmental cues. Specifically, *C. albicans* cells lacking Eri1, an endoplasmic reticulum localized protein that acts as a negative regulator of filamentation, did not require Ras1 for filamentation in the presence of HCO_3_−/CO_2_ [45]. Thus, our findings further emphasizes Ras1-independent activation of cAMP–PKA pathway for the induction of hyphae in *C. albicans*. In addition, another study showed the existence of cAMP-independent signals contributing towards the induction of hyphal formation, specifically in a pseudorevertant strain of *C. albicans* lacking Cyr1 [46]. Similarly, we show that iron-dependent filamentation can occur even in the absence of cAMP, as *C. albicans* lacking Cyr1 retained the ability to form hyphae under high-iron conditions (Figure 4A), and no cAMP was detected in high-iron *Δ/Δras1* cells despite hyphal formation (Figure 4B). However, deletion of downstream effectors such as Tpk2 or Efg1 abolished iron-induced filamentation in the *Δ/Δras1* and *Δ/Δcyr1* cells, indicating that the core PKA-dependent transcriptional machinery remains essential (Figure 4A).

In the cAMP-PKA signaling pathway, Bcy1 is the regulatory subunit that limits PKA activity under non-inducing conditions [13]. Multiple studies show that lower Bcy1 function or expression can encourage filamentation even without increased cAMP levels [13,47,48]. These findings suggest that changing Bcy1 activity helps *C. albicans* to adjust PKA signaling independent of cAMP levels in response to environmental signals. Our transcriptomic analysis also identified Bcy1 as an important factor in iron-dependent hyphal formation in the absence of active Ras1 cAMP signaling. Under high iron, *BCY1* expression was reduced in *Δ/Δras1* cells but not in the parental strain (Figure 5). Since Bcy1 normally inhibits Tpk2 activity without cAMP, its reduction likely releases this inhibition and allows PKA signaling to proceed [36]. Supporting this, overexpression of Bcy1 blocked iron-induced hyphal formation in *Δ/Δras1* cells (Figure 6). Although the present study demonstrates that high-iron conditions are associated with reduced *BCY1* expression and activation of filamentation, the molecular mechanisms linking iron signaling to *BCY1* regulation remain unclear. One possible explanation is that intracellular iron availability may influence transcriptional regulators, chromatin remodeling pathways, or stress-responsive signaling networks [23] that indirectly repress *BCY1* expression under high-iron conditions. Alternatively, iron-dependent metabolic adaptation or mitochondrial activity could alter PKA regulatory dynamics and thereby affect Bcy1 stability or expression. Future studies will therefore be necessary to define the upstream iron-responsive pathways that control *BCY1* regulation and determine how iron signaling interfaces with cAMP-PKA pathway components during morphogenetic switching.

**Figure 7:**
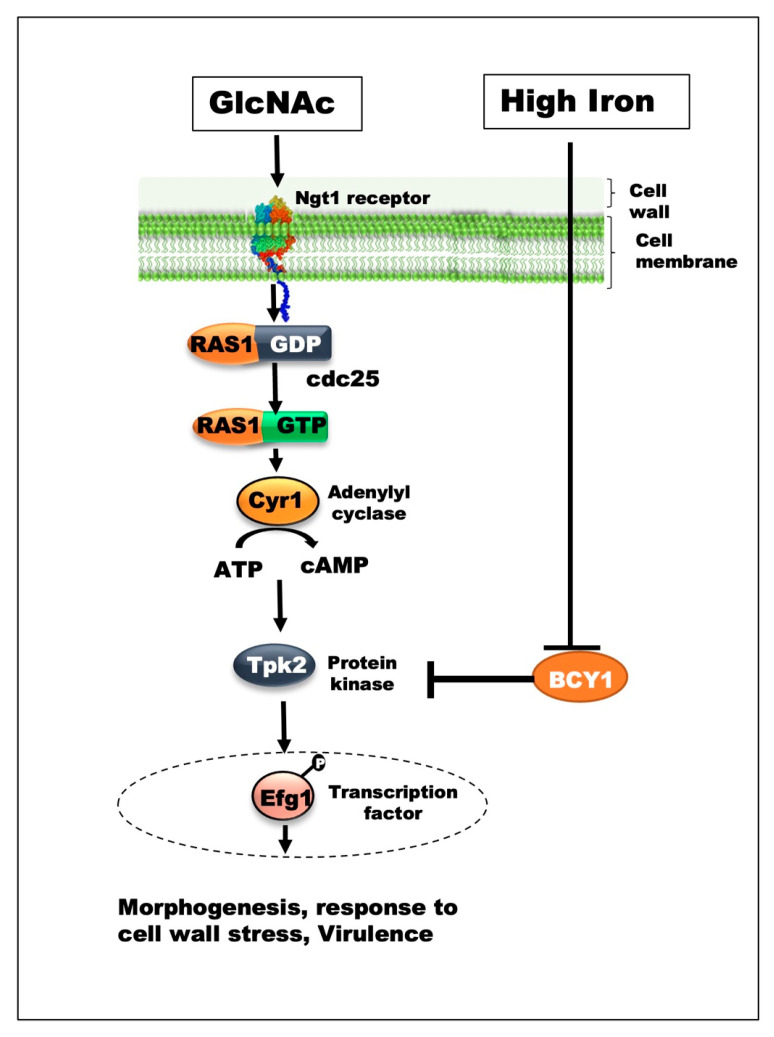
Model of high iron-induced Ras1-independent signaling in hyphal development. Schematic representation of signaling pathways regulating hyphal morphogenesis in *C. albicans*. Under canonical conditions, Ras1 activates Cyr1 to generate cAMP, which in turn activates PKA and drives hyphal growth. In contrast, under high iron, hyphal development can proceed independently of Ras1. Elevated iron levels suppress expression of Bcy1, the negative regulatory subunit of PKA, thereby relieving inhibition of Tpk2 and promoting PKA activation without support on the conventional Ras1–cAMP signaling cascade.

While *BCY1* overexpression abolished high iron-induced hyphal formation in the *Δ/Δras1* strain, we did not examine the effect of *BCY1* overexpression under standard iron conditions. Future studies evaluating the *Δ/Δras1* + *BCY1* overexpression strain across different iron environments will help determine whether *BCY1* specifically mediates iron-dependent hyphal induction or more broadly regulates filamentation independently of iron availability. Overall, our results show that iron promotes filamentation by lowering Bcy1 levels, thereby bypassing the need for Ras1-cAMP signaling (Figure 7). This mode of regulation is conceptually significant, as it positions iron not only as a metabolic cofactor but also as a direct modulator of signaling pathways governing fungal morphology.

From a broader perspective, these findings have important implications for antifungal strategies. Therapeutic approaches targeting upstream components of the Ras1–cAMP pathway may be insufficient to block filamentation in iron-rich host niches, where alternative activation mechanisms can compensate. This may partially explain the persistence and adaptability of *C. albicans* in diverse host environments. Future studies should focus on elucidating the molecular link between iron sensing and Bcy1 regulation, identifying the upstream iron-responsive factors involved and determining whether similar bypass pathways operate under other clinically relevant conditions. A deeper understanding of these adaptive signaling networks will be critical for the development of more effective antifungal interventions.

## Figures and Tables

**Table 1:** *C. albicans* strains used in the study.

Strain	Genotype	Reference
CAI4	Δura3::imm434/URA3	[25]
*Δ/Δcdc25*	Δura3::λimm434/ura3::λimm434arg4::hisG/arg4::hisG his1::hisG/his1::hisG Δcdc25::HIS1/cdc25::ARG4	[26]
*Δ/Δras1*	Δura3::λimm434/Δura3::λimm434 Δras1::hisG/ras1::hisG::URA3	[27]
*Δ/Δcyr1*	Δura3::λimm434/Δura3::λimm434 Δcyr1::hisG::Δcyr1::hisG	[28]
*Δ/Δtpk1*	Δura3::λimm434/Δura3::λimm434/his1::hisG/his1::hisG arg4::hisG/arg4::hisG tpk1::ARG4/tpk1::URA3	[27]
*Δ/Δtpk2*	Δura3::λimm434/Δura3::λimm434/his1::hisG/his1::hisG arg4::hisG/arg4::hisG tpk2::ARG4/tpk2::URA3	[27]
*Δ/Δefg1*	Δura3::λimm434/Δura3::λimm434/efg1::hisG/efg1::hisA-hisG-ura3::λimm434/ura3::λimm434 efg1::hisG	[27]
*Δ/Δras1 + BCY1*	Δura3::λimm434/Δura3::λimm434 Δras1::hisG/ras1::hisG::URA3 ADH1/adh1::P_tet_-BCY1-6xHis	In this study

**Table 2:** Primers used in the study.

Primers	Sequence	Reference
Forward *BCY1*	CGTCGGTCGACATGTCTAATCCTCAACAGCAATTC	This study
Reverse *BCY1*	AGCCTAGATCTTTAGTGATGGTGATGGTGATGATGACCAGCAGTTGGGTCTTGA	This study
Forward RT- *BCY1*	TCCAGTAGCGAAGGGTCATC	This study
Reverse RT- *BCY1*	TTCGACGGAATGTCAAACGG	This study
Forward RT- *HWP1*	ATCAGCTCCTGCCACTGAAC	This study
Reverse RT- *HWP1*	TGGCAGATGGTTGCATGAGT	This study
Forward RT- *GAPDH*	GGGTTCAAGCTACTCATCTATC	This study
Reverse RT- *GAPDH*	CTGGTGGAGTAACCGTATTC	This study

## Data Availability

The original contributions presented in this study are included in the article/Supplementary Materials. Further inquiries can be directed to the corresponding authors.

## Supplementary Materials

The following supporting information can be downloaded at: https://www.mdpi.com/article/10.3390/jof12070459/s1.
