# Supplementary material for: Ras1-Independent High Iron-Mediated Hyphal Formation in Candida albicans
Source: J Fungi (Basel). 2026 Jun 23;12(7):459. doi: 10.3390/jof12070459 (PMC13412770; doi:10.3390/jof12070459)
Supplement: Supplementary file 1 [file jof-12-00459-s001.zip › jof-4306236-supplementary.pdf]

Supplementary Information for

Ras1-independent High Iron-mediated Hyphal Formation in *Candida albicans*

Deepak Parashar[1], Rishabh Sharma[1], Sumant Puri[1]*

AUTHOR AFFILIATIONS

[1]Oral Microbiome Research Laboratory, Temple University, Philadelphia, USA
*Corresponding author: Sumant Puri,

Oral Microbiome Research Laboratory, Kornberg School of Dentistry, Temple University, 3223 N Broad Street, Philadelphia, PA 19140. Tel:215.707.5984
E-mail: sumantpuri@temple.edu

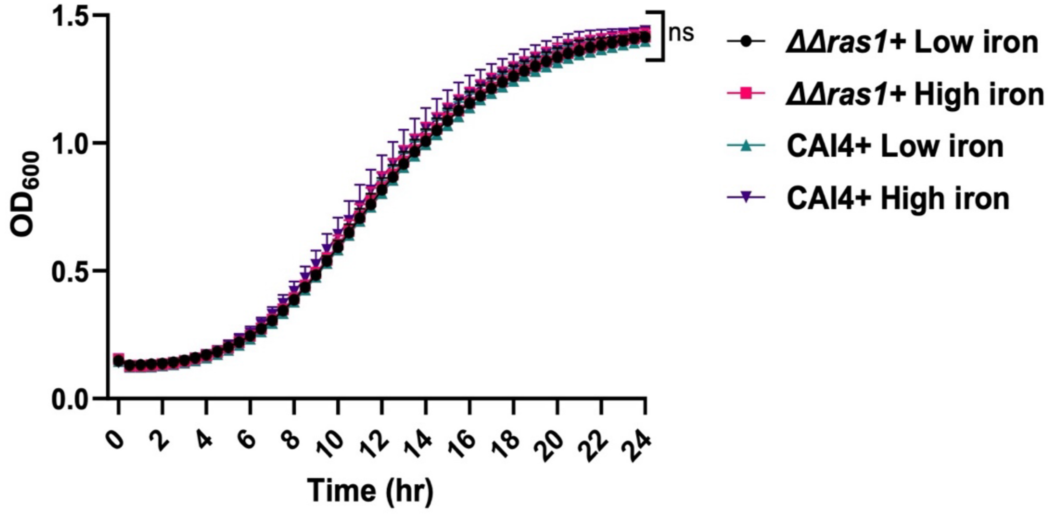

Figure S1. ***C. albicans*** **CAI4 and** ***Δ/Δras1*** **growth is unaffected at low and high iron.** *C. albicans* cells and *Δ/Δras1* cells were grown in YNB-glucose medium containing low iron (2 µM) and high iron concentrations (100 µM), and growth was monitored at OD_{600} over a period of 24 hours at 30°C. plates were shaken at the interval of 30 minutes for 5 Seconds. Results shown are of three biological repeats and ± standard errors of the means (SEM). (ns statically not significant).
